# Recurrence prediction in chronic subdural hematomas: a risk stratification score based on 118 consecutive patients

**DOI:** 10.1016/j.bas.2025.104286

**Published:** 2025-05-22

**Authors:** Francesco M.C. Lioi, Jon Ramm-Pettersen, Andrea Fratini, Gabriele Dentato, Giovanni Facchinetti, Niccolo Colella, Luigi Rosito, Elena Furno, Camilla Riva, Andrea G. Ruggeri, Luca D'Angelo, Antonio Santoro, Alessandro Frati

**Affiliations:** a Sapienza University of Rome, Rome, Italy; b Oslo University Hospital, Oslo, Norway

**Keywords:** Chronic subdural hematoma, Grading system, Score, Recurrence, Prediction

## Abstract

**Background:**

Chronic subdural hematoma (CSDH) is a frequent neurosurgical condition with a significant recurrence rate. Identifying patients at risk of recurrence is a topic of great interest to intercept this population in which to implement further therapeutic strategies.

**Objective:**

To develop and internally validate a risk-stratification score using preoperative radiological parameters to predict risk of CSDH recurrency. To establish a stratification approach based on easily available clinical and radiological variables.

**Methods:**

A retrospective analysis of 118 consecutive patients surgically treated for CSDH was conducted. Binary logistic regression was used to identify radiological preoperative predictors of recurrence, including Nakaguchi classification, cortical atrophy, and hematoma density.

**Results:**

A weighted scoring system has been developed and patients were stratified into three risk categories: low (0–2 points), moderate (3–5 points), and high (6–8 points). This grading system demonstrated a significant correlation with recurrence rates: 0 % in the low-risk group, 11.76 % in the moderate-risk group, and 25.71 % in the high-risk group. The model exhibited good discriminative ability in predicting the risk of CSDH recurrence.

**Conclusion:**

Hyperdense appearance, homogeneous or separated Nakaguchi-configuration and presence of cortical atrophy are predictive features of CSDH recurrence and can be incorporated into a score. The proposed grading system provides an effective preoperative tool for assessing CSDH recurrence risk, with a progressive increase in the recurrency rate between the three groups. This model enables personalized postoperative management, facilitating early identification of high-risk patients who may benefit from adjunctive treatments to reduce recurrence.

## Highlights

• A grading system incorporating Nakaguchi score, hematoma density, and cortical atrophy can help estimate the likelihood of chronic subdural hematoma (CSDH) recurrence.
• There are distinct risk groups, each representing progressively higher chances of recurrence.
• By recognizing those at greater risk, clinicians can tailor postoperative management strategies and potentially improve outcomes.

## Abbreviation list:

**AUC**: Area Under the Curve
**CSDH**: Chronic Subdural Hematoma
**CT**: Computed Tomography
**GCA**: Global Cortical Atrophy
**HU**: Hounsfield Units
**MMAE**: Middle Meningeal Artery Embolization
**OR**: Odds Ratio
**PACS**: Picture Archiving and Communication System
**RrR**: Recurrence requiring Reoperation
**ROC**: Receiver Operating Characteristic
**SD**: Standard Deviation

## 1 Introduction

Chronic subdural hematoma (CSDH) is an increasingly prevalent condition, particularly in the elderly population, and is characterized by a non-negligible recurrence rate. Recurrence of CSDH is a significant event that leads to increased morbidity in an already vulnerable patient group. It is considered a sentinel health event, comparable to stroke, heart disease, hip fracture, and hospitalization for cancer (Dumont et al., 2013). The recurrence of CSDH negatively impacts patients' quality of life and incurs substantial healthcare costs due to rehospitalization and the associated medical and surgical treatments. Formally, CSDH recurrence was defined by Laumer et al. as: (1) an increase, recurrence, or lack of improvement of an initial neurological deficit, or the presence of new symptoms attributable to a corresponding CSDH; (2) an increase in hematoma size with or without neurological deterioration; or (3) a persistent or recurrent severe headache with corresponding findings on CT scan (Laumer et al., 1989). Recurrence may occur within a short time frame or even at a later stage. Reported recurrence rates in the literature vary widely (Kolias et al., 2014; Liu et al., 2014), but contemporary consensus estimates the reoperation rate between 2.5 % and 33 % of cases (Stanišic and Pripp, 2017). The pathophysiological factors influencing recurrence can be patient-specific, such as poor brain elasticity and consequent low parenchymal re-expansion following hematoma evacuation (Kung and Lin, 2020), patient predisposition to rebleeding (e.g., coagulopathies or anticoagulant use) (Jeong et al., 2014), or the medical therapy administered (Yu et al., 2022). Additionally, surgical factors, including the type of intervention performed (Nouri et al., 2021; Al- et al., 2023), perioperative management strategies, and the use of drainage (including its direction) (Santarius et al., 2009), play a role. Drainage type (subdural or subperiosteal) (Soleman et al., 2019) and duration of its use before removal (Kolias et al., 2014; Adeolu and Rabiu, 2012) have also been associated with recurrence risk. Complex inflammatory reactions, angiogenesis, local coagulopathy, and recurrent micro-hemorrhages are suggested to be key mechanisms in hematoma formation, expansion, maintenance, and recurrence, justifying the use of pharmacological agents such as dexamethasone, statins, or antifibrinolytics (tranexamic acid), which interfere with these metabolic, inflammatory, and coagulative cascades (Albalkhi et al., 2024; Georgountzos et al., 2023; He et al., 2021). Radiological characteristics, particularly the internal architecture of CSDH, are considered closely related to recurrence in surgically treated patients. Nakaguchi et al. categorized radiological features most frequently associated with recurrence (Nakaguchi et al., 2001). The strongest predictors include hyperdense or isodense hematomas with laminar or separated structures. Additionally, a postoperative CSDH cavity volume exceeding 200 mL (Stanišic and Pripp, 2017) has been associated with a higher recurrence risk. CSDH recurrence remains a critical issue in neurosurgical practice, often necessitating reoperation. The development of scoring systems for recurrence risk prediction, integrating radiological and clinical factors, enables improved patient stratification. This facilitates personalized follow-up schedules, decision-making regarding the extent of postoperative drainage, and identification of candidates for adjunctive therapies such as middle meningeal artery embolization (Shen et al., 2019; Liu et al., 2024; Link et al., 2019). By integrating clinical and imaging data, these predictive tools enhance patient outcomes and optimize resource allocation, directing preventive treatments toward high-risk patients. Although a universally accepted grading system for predicting postoperative recurrence requiring reoperation (RrR) in CSDH patients is lacking, several scoring systems have been proposed. The aim of this study is to delineate the most significant radiological and clinical factors associated with recurrence.

## 2 Materials and methods

### 2.1 Objective

We analyzed clinical and radiological parameters predictive of chronic subdural hematoma recurrence to preoperatively identify patients at risk of recurrence.

### 2.2 Study design

This retrospective, single-center study was conducted at the Department of Neurosurgery, Policlinico Umberto Primo Hospital in Rome, ‘affiliated with the University of Rome ‘La Sapienza.’ Medical records from January 2020 to December 2024 were analyzed to identify patients diagnosed with chronic subdural hematoma who were surgically treated and followed over time to detect recurrence, with subsequent analysis to determine predictors of this event.

### 2.3 Inclusion criteria

Adults aged 18 years or older with unilateral or bilateral hematoma treated surgically were included. Surgical indications were based on clinical and radiological criteria, including focal neurological deficits, altered mental status, hematoma thickness (>10 mm), or midline shift (>5 mm). All patients had preoperative CT scans and underwent surgery with a single burr-hole under local anesthesia, followed by cavity irrigation and closed-system drainage for ≥24 h. In accordance with the surgical practice routinely adopted at our center, we systematically used an irrigation protocol with isotonic saline solution (0.9 % NaCl), adjusted to body temperature (37 °C), for every patient. The choice of using a solution with these characteristics may positively influence coagulation and the solubility of chronic subdural hematoma (CSDH) compared to irrigation fluids at room temperature (Bartley et al., 2017, 2023). Moreover, isotonic saline appears to be the most readily available alternative, given its widespread use in nearly all neurosurgical procedures. Regarding the type and placement of the drainage system, our strategy consisted in positioning a passive and anteriorly-pointing subdural closed drainage system. This approach reflects the standard practice at our center and was adopted uniformly for all patients included in the study. This allowed us to focus on the investigation of recurrence within a surgically homogeneous population, thereby minimizing potential confounding factors related to drainage strategy. A CT scan was performed on the first postoperative day, and drainage removal was decided case-by-case, never exceeding 72 h. Patients were mobilized after drainage removal and underwent follow-up for at least 6 months. Those not meeting these criteria were excluded.

### 2.4 Data acquisition

**Fig. 1:**
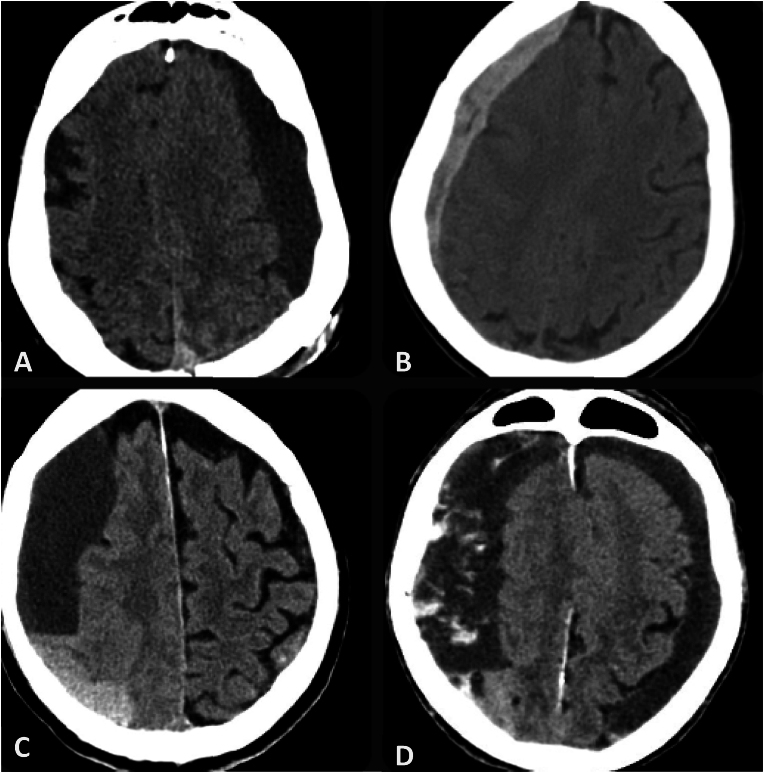
Nakaguchi classification of CSDH. A: homogeneous, B: laminar, C: separated, D: trabecular.

Once the study population was defined, data were collected for each patient on sex, age, etiology (traumatic or spontaneous), and hematoma laterality. Radiological characterization included preoperative and postoperative assessment of qualitative (Nakaguchi scale, cortical atrophy), semiquantitative (hematoma density), and quantitative parameters (midline shift, hematoma volume, pneumocephalus volume). Each case was assigned to one of 4 classes based on the radiological pattern as described by Nakaguchi (Fig. 1): homogeneous, laminar, separated and trabecular. Accordingly, the homogeneous type is characterized by a regular density pattern (which can be hyperdense, isodense, or hypodense) that remains consistent throughout its extent. The laminar type is distinguished by a linear hyperdense subcompartment located along the inner membrane of the hematoma. The separated type exhibits two distinct densities, with a visible level separating the denser, dependent component from the less dense one. These two components do not homogenize with normal head motions. Lastly, the trabecular type features internal hyperdense septations, resulting from fibrotic processes and the reorganization of the internal membranes within the hematoma. Cortical atrophy was evaluated using the Global Cortical Atrophy (GCA) scale, originally described by Pasquier et al. This qualitative scale assesses cortical atrophy based on the widening of sulci and ventricular enlargement, scoring regions of the brain from 0 (no atrophy) to 3 (severe atrophy). For the purpose of this study, we adapted the GCA scale to dichotomize patients into two groups: absence of atrophy (GCA score = 0) and presence of atrophy (GCA score ≥1). This binary classification was chosen to simplify data interpretation and to align with our study purposes. While the original scale allows for a progressive gradation of cortical atrophy, the dichotomous approach enabled a clear differentiation between patients with and without atrophic changes, facilitating statistical analysis. Hematoma density was defined on the basis of attenuation values expressed in Housfield units (HU) as hypodense (20–30 HU or less), isodense (30–40 HU) or hyperdense (>40–50 HU). The remaining quantitative parameters were assessed by one of the authors (G.D.) using a semi-automated computer-assisted quantification program integrated into our hospital's PACS system (Sectra, Sectra AB, Linköping, Sweden).

### 2.5 Endpoints

Patients included in this study followed a structured follow-up protocol comprising clinical evaluations and radiological assessments, including outpatient visits and CT scans conducted one month after surgery. Beyond the first month, follow-up was limited to clinical evaluations in the absence of complications. Our study focuses on patients with recurrence of chronic subdural hematoma (CSDH), using both clinical and radiological criteria. Clinical recurrence was defined as the reappearance or worsening of neurological symptoms related to the hematoma, requiring rehospitalization or additional treatment in a patient who had previously shown improvement after the initial surgery. Radiological recurrence was defined as the reformation or an increase in the thickness or volume of the hematoma observed during follow-up evaluations after the initial treatment.

### 2.6 Statistical methods

Descriptive statistics was used to summarize demographic and clinical features. A predictive score was developed using a binary logistic regression model to identify significant predictors of recurrence in chronic subdural hematoma (CSDH). Variables with a p-value <0.05 were included in the final model, and points were assigned to each significant item proportionally to its odds ratios. Based on the total score, patients were stratified into three risk categories: low, moderate, and high risk. The thresholds for these categories were defined to reflect clinically meaningful risk gradients. The predictive score was validated through statistical analysis to assess its ability to stratify patients into clinically meaningful risk categories. Model performance was evaluated using receiver operating characteristic (ROC) curve analysis, with the area under the curve (AUC) as the primary measure of its discriminative ability. The association between risk categories and recurrence rate was assessed using chi-square tests, and odds ratios were calculated to quantify differences between risk levels. All analyses were conducted using Python (pandas, sklearn, matplotlib) and R for visualization.

### 2.7 Ethical considerations

All procedures involving human participants complied with the ethical standards of the institutional and national research committees, as well as the 1964 Declaration of Helsinki and its amendments. Informed consent was obtained from all patients or their next of kin. This retrospective study used anonymized data, and formal ethical approval was not required as it did not alter diagnostic or therapeutic practices.

## 3 Results

### 3.1 Descriptive statistics

Our population consisted of 118 patients with a mean age of 74 years (SD = 11), ranging from 40 to 93 years. This cohort included 91 males (77 %) and 27 females (23 %). Regarding etiology, 52 % of cases (62 patients) were of traumatic origin, while 48 % (56 patients) were spontaneous. Lateralization showed an equal distribution of right-sided and left-sided hematomas, each observed in 39 % of patients (47 cases), while 20 % (24 cases) presented with bilateral hematomas. The most common clinical presentation was focal neurological deficit, observed in 35 patients (29.7 %), followed by a combination of focal deficit and cognitive decline in 30 patients (25.4 %), and focal deficit with subsequent falling episodes in 21 patients (17.8 %). Other presentations included combinations of focal deficit, cognitive decline, and falling episodes in 11 patients (9.3 %), isolated cognitive decline in 8 patients (6.8 %), and isolated falling episodes in 6 patients (5.1 %). Less frequent combinations accounted for the remaining 5.9 % of cases. According to Markwalder's scale, the majority of patients were classified as grade 1 with 60 patients (51 %), followed by grade 2 with 40 patients (34 %) and grade 3 with 18 patients (15 %). This distribution indicates that more than half of the patients presented with mild or moderate symptoms (grade 1), with a progressively smaller proportion of patients exhibiting more severe presentations on the scale. Hematoma density was predominantly hypodense in 58 % of patients (70 cases), with hyperdense and isodense densities observed in 25 % (29 cases) and 16 % (19 cases), respectively. According to Nakaguchi scale, 38 % of hematomas (45 cases) were homogeneous, 35 % (41 cases) were trabecular, 26 % (31 cases) were laminar, and 7 % (8 cases) were separated. Anticoagulant or antiaggregant therapy was administered to 82 patients (71.9 %), while 32 patients (28.1 %) did not receive such therapy. Preoperative hematoma volume averaged 85.4 mL (SD: 42.3), ranging from 35 to 210 mL, while postoperative residual hematoma volume had a mean of 26.7 mL (SD: 16.8), ranging from 0 to 75 mL. Mean pneumocephalus volume was 5.1 mL (SD: 4.3), with a range of 0–20 mL. 15 patients (12.7 %) experienced recurrence of chronic subdural hematoma as previously defined. Of those, 10 (66.7 %) required a reintervention. Table 1 shows relevant clinical and radiological parameters of our study population.

**Table 1:** Clinical and radiological parameters.

**Parameter**	**Values**
**Sex**	91 M (77 %), 27 F (23 %)
**Age**	74 (range 40–93)
**Use of anticoagulants**	82 (71.9 %) Yes, 32 (28.1 %) No
**Etiology**	62 (52 %) Trauma, 56 (48 %) Spontaneous
**Markwalder's scale**	Grade I: 60 (51 %), Grade II: 40 (34 %), Grade III: 18 (15 %)
**Density**	Hypodense: 70 (58 %), Isodense: 19 (16 %), Hyperdense: 29 (25 %)
**Nakaguchi scale**	Homogeneous: 45 (38 %), Trabecular: 41 (35 %), Laminar: 31 (26 %), Separated: 8 (7 %)

### 3.2 Development and validation of a grading system

Our grading system was developed through a binary logistic regression analysis aimed at identifying predictors of recurrence in chronic subdural hematomas. Variables have been dichotomized to simplify the analysis. Accordingly, within the density variable, we compared hyperdense hematomas against isodense and hypodense ones grouped together. For the Nakaguchi classification, we considered homogeneous and separated types as one category, contrasted with the laminar and trabecular types. Cortical atrophy was classified as either present or absent. Homogeneous or separated-type hematomas according to Nakaguchi type (p = 0.032), presence of cortical atrophy (p = 0.008), and hematoma hyperdense structure (p = 0.001) were identified as significant predictors. Points were assigned proportionally to the odds ratios of these variables (Table 2): cortical atrophy, with the highest odds ratio (OR = 25.20), was assigned 5 points; hematoma density (OR = 10.39) was assigned 2 points; and Nakaguchi classification (OR = 4.86) was assigned 1 point. The total score ranged from 0 (no risk factors) to 8 (all risk factors present), allowing for stratification into low (0–2), moderate (3–5), and high (6–8) risk categories. We applied our grading system to stratify patients into three risk categories: low risk (32 patients), moderate risk (51 patients), and high risk (35 patients). Recurrence rates increased progressively across these categories, with no recurrences in the low-risk group (0 %), 11.76 % in the moderate-risk group, and 25.71 % in the high-risk group (Table 3). The model demonstrated good discriminative performance, as indicated by an Area Under the Curve (AUC) of 0.736. Stratification validity was further supported by odds ratios, showing a fourfold increased risk of recurrence between the moderate- and low-risk groups (OR = 4.0, 95 % CI: 1.58–10.15) and between the high- and moderate-risk groups (OR = 4.0, 95 % CI: 1.58–10.15) (Table 4). The greatest risk difference was observed between the high- and low-risk groups, with an odds ratio of 16.0 (95 % CI: 6.25–41.15).

**Table 2:** Score development.

Parameter	Odds Ratio	Points
Cortical atrophy	25.20	5
Hematoma density	10.39	2
Nakaguchi classification	4.86	1
Total score	–	8

**Table 3:** Risk categories for CSDH-recurrency.

Risk categories	Points	Recurrence rate
Low	0–2	0 %
Moderate	3–5	11.76 %
High	6–8	25.71

**Table 4:** Odds ratio for specific comparisons between risk categories.

Comparison	Odds Ratio	95 % CI Lower	95 % CI Upper
High vs Moderate	4.00	1.58	10.15
Moderate vs Low	4.00	1.58	10.15
High vs Low	16.00	6.25	41.15

## 4 Discussion

Chronic subdural hematoma is a frequently encountered neurosurgical condition, particularly in the elderly population. Although surgical evacuation remains the consolidated treatment, recurrence remains a problem whose extent is significant, ranging from 10 to 33 % depending on the technique used and the characteristics of the patient (Kung and Lin, 2020). Recurrence inevitably entails consequences in terms of morbidity, mortality as well as additional costs related to rehospitalization. Identifying patients who are at risk of recurrence therefore represents a question of great interest as it explores the possibility of optimizing perioperative and postoperative treatment strategies aimed at reducing the need for reoperation. Our work investigates a consecutive series of 118 patients with key risk factors for CSDH recurrence and explores the possibility of developing a predictive scoring system to preoperatively identify patients who are at risk of recurrence.

### 4.1 Risk factors for CSDH recurrence

Several studies have focused on the study of factors associated with CSDH recurrence. These can be divided into patient-related factors, radiological factors related to imaging characteristics, and purely surgical factors. Among the patient-related factors, cerebral atrophy and aging figure prominently. These conditions, common in elderly patients, lead to the configuration of an enlarged subdural space post-evacuation, and reduced parenchymal elasticity leads to limited brain re-expansion and an increased recurrence risk (He et al., 2021). Certainly, medical comorbidities also play a role. Conditions such as liver disease, coagulation disorders, and the presence of diabetes mellitus have been linked to an increased risk of recurrence (Kolias et al., 2014). Last but not least, the use of anticoagulants or antiplatelets are well-documented risk factors for hematoma recurrence (Kung and Lin, 2020).

### 4.2 Radiological features

The radiological factors associated with recurrence studied in the literature are both qualitative and quantitative. The density and morphology of the CSDH are among the main ones (Kung and Lin, 2020; Link et al., 2019). Oslo group focused on the quantitative study of the hematoma volume, demonstrating how a residual hematoma greater than 200 mL postoperatively can be associated with a higher recurrence rate. According to Stanišić et al. a residual hematoma cavity of more than 80 mL on the first postoperative day increases the likelihood of recurrence (Stanišic and Pripp, 2017). Unfortunately, with our data we were not able to repeat an association of this kind. Certainly the presence of highly vascularized membranes in the structural context of the CSDH has been strongly associated with the recurrence risk, as demonstrated by Chen et al. who focused on contrast imaging for the characterization of this aspect (Al- et al., 2023).

### 4.3 Surgical factors

Several factors widely discussed in the literature related to surgery may be associated with an increased risk of recurrence. A detailed analysis of these is beyond the scope of this work. Certainly the usefulness of reducing the risk of recurrence through the positioning of a surgical drain is a consolidated acquisition in the literature as demonstrated by the classic study by Santarius et al. (2009). The positioning of the drain in the frontal region also seems to significantly reduce the recurrence rates compared to other types of drainage orientation (Kolias et al., 2014). Several studies also support the relative equipoise between subdural or subperiosteal drain insertion concerning recurrence, reoperation rate or clinical outcome (Kamenova et al., 2020). Regarding the duration of drainage, a longer postoperative drainage period is associated with a lower recurrence rate compared to shorter duration (Hjortdal et al., 2024), especially in cases with larger residual volume after surgical evacuation (Stanišic and Pripp, 2017). The presence of intracranial air after surgery is not least a well-established risk factor for recurrence, underlining the importance of developing air replacement techniques using normal saline irrigation.

### 4.4 Rationale for developing a CSDH-recurrence predictive risk score

The interest in identifying the subgroup of patients who are at higher risk of developing CSDH recurrence is linked to the scenario of early interception of the recurrency with the aim of implementing measures and therapeutic strategies aimed at reducing its clinical burden. The practical utility of developing predictive scores is therefore to guide clinicians toward more aggressive interventions that can vary from a prolonged timing of drainage, adjunct pharmacological treatments such as statins and tranexamic acid (Iorio et al., 2016) up to middle meningeal artery embolization (MMAE) (Liu et al., 2024). In this sense, the interest in developing probabilistic scores capable of estimating the likelihood of recurrence starting from clinical-radiological elements that are easy to extract from the daily clinical context becomes evident. Our score is placed precisely within this framework and presents as a strong point the easy applicability on the basis of preoperative radiological parameters that are easy to identify.

### 4.5 Limitations and future perspectives

This study has several limitations. First, its retrospective and single-center design may limit the implementation of the findings to other populations or healthcare settings. The relatively small sample size, with only 15 recurrence cases, might reduce the statistical power to identify all potential predictors of recurrence. Additionally, the minimum follow-up period of 6 months may not capture late recurrences, particularly in chronic subdural hematomas. Methodologically, dichotomizing variables such as the GCA scale could have led to a loss of information and reduced the sensitivity of the model, while semi-automated quantification tools might introduce inter-operator variability or software-related errors. The proposed grading system, although internally validated, lacks external approval on independent cohorts, raising questions about its applicability in broader contexts. Furthermore, the cut-offs used to define risk categories were not extensively justified, potentially impacting the clinical relevance of the stratification. Lastly, as a retrospective study, it may be subject to biases in data collection and interpretation, despite adherence to ethical guidelines. Future research with prospective designs, larger cohorts, and external validation is needed to confirm these findings and enhance the reliability of the proposed model. One of the perspectives of future investigation will be to study the validation of this grading system with other centers and once this is done, select the patients at highest risk of recurrence thus stratified to direct them to additional treatments aimed at reducing the risk of recurrence such as the use of tranexamic acid or embolization of the middle meningeal artery.

## 5 Conclusions

Our proposed grading system identified Nakaguchi homogeneous or separated-type hematomas, presence of cortical atrophy and hematoma hyperdense structure as significant predictors of CSDH recurrence. We stratified patients into low-, moderate-, and high-risk categories, demonstrating a progressive increase in recurrence rates (0 %, 11.76 %, and 25.71 %, respectively). Our model showed good discriminative performance and strong risk differentiation, with a 16-fold increased recurrence risk in high-risk patients compared to low-risk ones. This system enables an easy and preoperative risk assessment for CSDH recurrency, with a potential for selecting patient for optimization of further postoperative management strategies.

## Funding

This research did not receive any specific grant from funding agencies in the public, commercial, or not-for-profit sectors.

## Declaration of competing interest

The authors declare that they have no known competing financial interests or personal relationships that could have appeared to influence the work reported in this paper.
